# Transcriptome analysis of molecular mechanisms responsible for light-stress response in *Mythimna separata* (Walker)

**DOI:** 10.1038/srep45188

**Published:** 2017-03-27

**Authors:** Yun Duan, ZhongJun Gong, RenHai Wu, Jin Miao, YueLi Jiang, Tong Li, XiaoBo Wu, YuQing Wu

**Affiliations:** 1 Institute of Plant Protection, Henan Academy of Agricultural Sciences, Key Laboratory of Crop Pest Control of Henan Province, Key Laboratory of Integrated Pest Management on Crops in Southern Region of North China, Zhengzhou 450002, China

## Abstract

Light is an important environmental signal for most insects. The Oriental Armyworm, *Mythimna separata*, is a serious pest of cereal crops worldwide, and is highly sensitive to light signals during its developmental and reproductive stages. However, molecular biological studies of its response to light stress are scarce, and related genomic information is not available. In this study, we sequenced and *de novo* assembled the transcriptomes of *M. separata* exposed to four different light conditions: dark, white light (WL), UV light (UVL) and yellow light (YL). A total of 46,327 unigenes with an average size of 571 base pairs (bp) were obtained, among which 24,344 (52.55%) matched to public databases. The numbers of genes differentially expressed between dark vs WL, dark vs UVL, dark vs YL, and UVL vs YL were 12,012, 12,950, 14,855, and 13,504, respectively. These results suggest that light exposure altered gene expression patterns in *M. separata*. Putative genes involved in phototransduction-fly, phototransduction, circadian rhythm-fly, olfactory transduction, and taste transduction were identified. This study thus identified a series of candidate genes and pathways potentially related to light stress in *M. separata*.

The Oriental Armyworm, *Mythimna separata* (Walker), also known as the Northern Armyworm or Rice Ear-cutting Caterpillar, is a highly destructive agricultural pest^1^, mainly distributed in Asia, Europe, and Oceania^2^,^3^,^4^,^5^,^6^. The larvae of this pest cause extensive damage to a range of agricultural plants such as wheat, maize and rice, and cause significant economic damage to corn crops in many parts of the world^6^,^7^,^8^. *M. separata* has been responsible for serious periodic damage to cereals in China since 1950^8^, with some outbreaks resulting in complete crop loss. Recent outbreaks of this pest have been recorded in many areas of China, especially in Jilin, Liaoning, Heilongjiang, Hebei and Shanxi, and its damage has posed a severe threat to corn production in this country^9^,^10^,^11^,^12^. Recent outbreaks of *M. separata* have also been reported in Indian and Korean^4^,^13^.

Circadian rhythms refer to physical, mental, and/or behavioural changes that follow an approximately 24-h cycle, responding primarily to the natural light-dark cycle in an organism’s environment^14^. Circadian rhythms reflect an evolutionarily old process and thus influence many organisms, including insects^14^,^15^, and such rhythms have been shown to affect many behaviours regulated by light^16^,^17^. Light, as an important environmental signal, may affect e.g. metabolism, reproduction, development, vision and locomotion^18^,^19^,^20^, though these effects are complex and vary among species^20^,^21^,^22^. Furthermore, exposure of animals to irregular light environments may alter their visual environment and thus their vision and related activities^23^,^24^,^25^.

Most insects, especially nocturnal insects, are sensitive to and attracted to artificial light^19^. In view of this phenomenon, several kinds of light traps have been used to monitor or reduce the populations of nocturnal insects, particularly noctuid moths^19^,^26^. Artificial light can alter the habitat of nocturnal insects and disturb their normal behaviours, including flight, orientation, dispersal, migration, communication, foraging, mate recognition, oviposition, eclosion, and daily activity rhythms^22^,^23^,^24^,^25^, as well as affecting reproduction and populations of nocturnal insects by altering the night-time environment^27^,^28^. As for other environmental factors, insects respond to artificial night-time light with an array of behaviours, and biochemical and genetic changes^23^,^27^,^29^.

*M. separata* is a nocturnal insect that is mainly active at night^23^. It exhibits an array of nocturnal behaviours such as flight, dispersal, migration, mate recognition, oviposition and eclosion, which mainly occur before sunrise and after sunset, thus demonstrating circadian rhythms, and also being regulated by light^2^,^23^. Adult *M. separata* have highly sensitive visual systems and are almost invariably strongly attracted to light^30^. Compound eyes composed of ommatidia are the principle visual organs, and can discriminate wavelengths in the range from ultraviolet to yellow, but not red^31^. Artificial light has been shown to affect multiple aspects of this species^32^,^33^,^34^; its development and reproduction may be affected by photoperiod, and by yellow and UV-A light^32^,^33^,^34^, while its flight capability may also be affected by photoperiod^32^. However, the effects of artificial light at the molecular level have not yet been reported from studies on *M. separata*.

Some studies have investigated the relationships between light and behavioural and physiological changes in insects^35^,^36^. However, understanding of the molecular responses of insects to light is based on a small number of species^37^,^38^,^39^,^40^,^41^,^42^, and has mainly focused on two pathways of circadian rhythm and phototransduction. Several genes related to circadian rhythm have been identified in *Drosophila melanogaster*^16^, *Anopheles gambiae*^37^ and *Ptomaphagus hirtus*^38^, and genes related to phototransduction have been found in *D. melanogaster*^39^,^40^,^41^ and *Periplaneta americana*^42^. Genomic information related to artificial light-induced changes in signalling and physiological processes, and the activation of complex regulatory networks (including transcriptional and post-transcriptional regulators) is currently limited^37^,^38^, and molecular biological studies of the responses of nocturnal insects to light stress are also scarce^38^,^41^.

Next-generation sequencing technologies provide a valuable tool for analysing transcriptome complexity and gene regulation^43^,^44^, and can also offer powerful insights into the genetic basis of the response of nocturnal insects to artificial light. In this study, we treated *M. separata* with artificial light conditions (dark, and white, UV and yellow light) that differed from natural sunlight conditions, and which may therefore affect the circadian rhythm or daily fluctuations of this species. We then adopted a mixed sampling strategy to create a moth-transcriptome database, and analyzed differential gene expression profiles from moths exposed different wavelengths of light. The main objectives were: (1) to construct a transcriptome dataset and annotate the generated genes; (2) to perform differentially expressed gene (DEG) analysis to identify genes responsive to light exposure; and (3) to characterize the light-altered biological processes and pathways and their associations with the sensory system, environmental adaptation, and other major biological functions. This is the first data set for the *M. separata* transcriptome related to light, and this resource will thus provide the basis for further powerful genetic and genomic analyses of this important pest species.

## Results

### Illumina sequencing and *de novo* assembly

**Figure 1:**
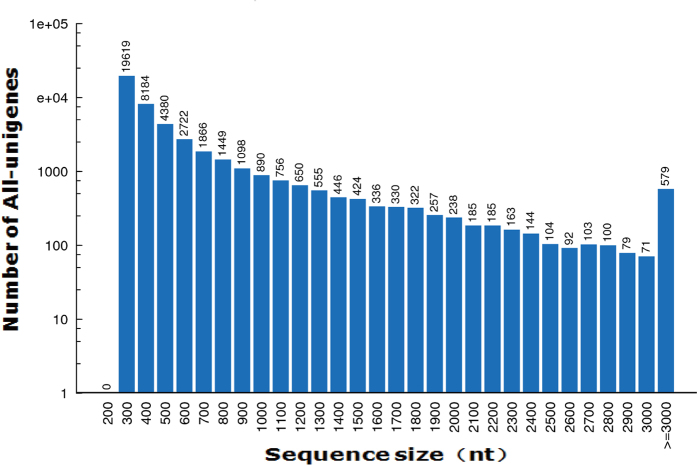
Distribution of transcript lengths from 200 to 11,534 nucleotides.

Four cDNA libraries were prepared from *M. separata* exposed to dark (dark), white light (WL), UVA light (UVL), and yellow light (YL), respectively, and subjected to Illumina deep sequencing. Illumina paired-end sequencing generated a total of 235.0 million raw reads. After cleaning and quality checks, 212.5 million clean reads (15.08 Gb) were obtained, with an average of 53.1 million reads (~3.8 Gb) per sample. The dark, WL, UVL, and YL cDNA libraries generated 54,402,458, 55,437,108, 50,414,324 and 52,197,036 clean reads, respectively (Table 1). The percentages of Q20 sequences (with an error probability of 0.01; high-quality indicator) in the four libraries were 98.12%, 97.04%, 96.93%, and 98.23%, respectively. These clean reads were *de novo* assembled into unigene sequences using Trinity software. These unigenes, encompassing 26,465,921 nucleotides (nt), were finally summarized into 46,327 All-unigenes, including 11,130 clusters and 35,197 singletons, with N50 at the length of 795 base pairs (bp) and an average length of 571 bp (Table 2). Among these All-unigenes, 6175 were exclusive to the WL group, and 6916, 6935, and 2563 were exclusive to the dark, UVL, and YL groups, respectively. The length distributions of All-unigenes are shown in Fig. 1. The most abundant unigenes were <300 bp (>19,000) and the least abundant were ≥3000 bp (579). Sequences ≥3000 bp were grouped together. Regarding the consensus sequences, many unigenes (69.50%) were 200–500 bp long, 13,906 were >500 bp, 5881 (12.69%) were >1000 bp, and 3.85% were >2000 bp. The number of sequences decreased as the length increased.

### Functional annotations and open reading frame predictions of All-unigenes

**Figure 2:**
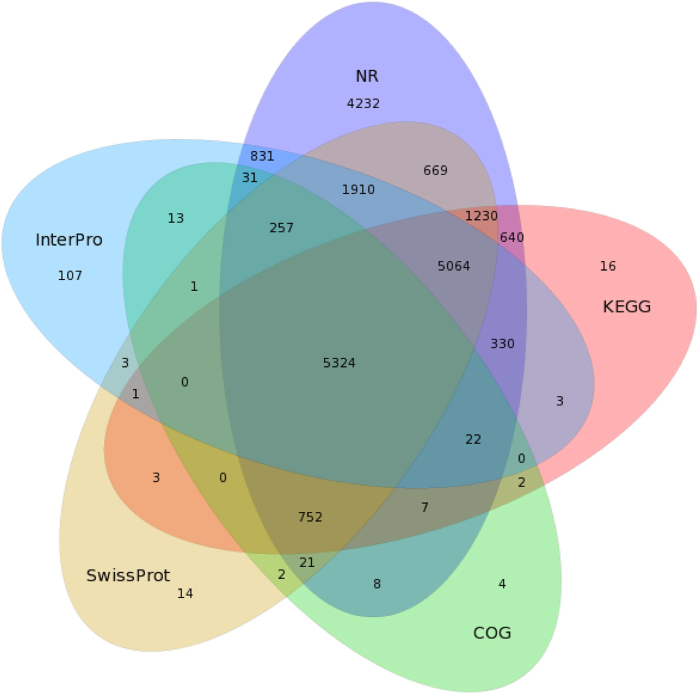
Venn diagram of shared and unique unigenes in *M. separata*. Annotation according to the nr, nt, Swiss-Prot, KEGG, COG, GO, and Interpro databases.

All-unigenes were searched for annotations against different databases. Assembled unigenes were subjected to blastx (BLAST, the basic local alignment search tool) alignment (E value < 1e-5) and several protein databases (including nr, Swiss-Prot, Kyoto Encyclopedia of Genes and Genomes (KEGG), Clusters of Orthologous Groups of Proteins (COG), and Interpro). In total, 21,328 (46.00%), 17,610 (38.01%), 15,251 (32.92%), 13,394 (28.9%), 6444 (13.91%), 10,621 (22.93%) and 13,897 (30.00%) All-unigenes were annotated to the nr, nt, Swiss-Prot, KEGG, COG, Gene Ontology (GO), and Interpro databases, respectively (Fig. 2, Supplementary File 1). A total of 24,344 (52.55%) unigenes were annotated, and the nr database had the most matches.

**Figure 3:**
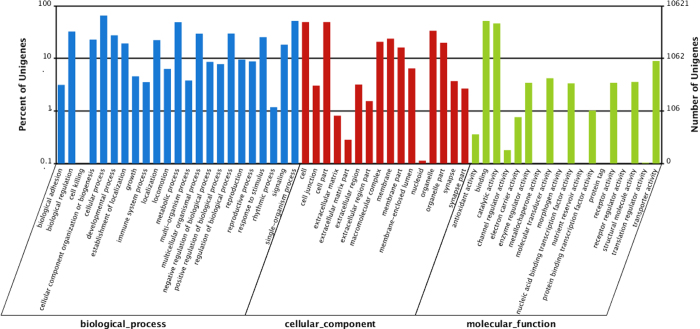
Gene ontology classification analysis of All-unigenes. The left axis indicates the percentage of All-unigenes in the main category within the specific category, and the right y-axis indicates the number of All-unigenes in a category.

GO assignments were used to classify the functions of All-unigenes based on nr annotation using Blast2GO. Among All-unigenes with significant hits in the nr database, 10,621 were categorized into 57 sub-categories belonging to three main GO categories: biological process, cellular component, and molecular function (Fig. 3, Supplementary Table S1), including 23, 16, and 18 sub-categories, respectively. The top 10 sub-categories were cellular process (6929), single-organism process (5482), binding (5475), cell (5220), cell part (5220), metabolic process (5183), catalytic activity (4897), organelle (3554), biological regulation (3442), and regulation of biological process (3150). In GO analysis, developmental process (2919), localization (2363), locomotion (668), reproduction (1008), reproductive process (931), response to stimulus (2693), and rhythmic process (125) were found in biological process.

**Figure 4:**
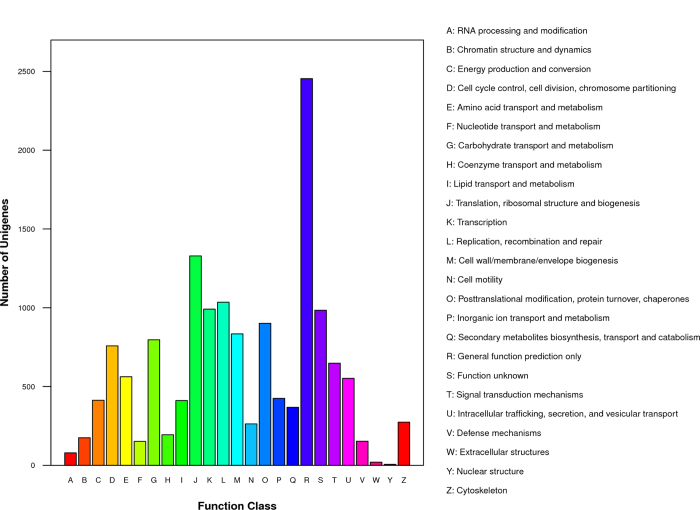
Distribution of All-unigenes among COG functional classifications.

To evaluate the completeness of these transcriptome libraries and the effectiveness of our annotation process, we searched the annotated sequences for genes involved in COG classifications. The 6444 assembled All-unigenes were divided into 25 categories (Fig. 4, Supplementary Table S2). The top five categories were translation, ribosomal structure and biogenesis (20.62%), replication, recombination and repair (16.06%), function unknown (15.28%), posttranslational modification, protein turnover, chaperones (13.98%), and carbohydrate transport and metabolism (12.37%), while extracellular structures (20, 0.31%) and nuclear structure (2, 0.09%) were the least represented.

To identify the biological pathways that are active in *M. separata*, we mapped 21,328 annotated sequences to the reference canonical pathways in the KEGG database. A total of 13,394 All-unigenes were assigned to 258 KEGG pathways belonging to six KEGG categories (Supplementary Tables S3, S4): metabolism (6694 unigenes, 21.7%), genetic information processing (3115, 10.9%), cellular processes (2982, 10.4%), human diseases (7096, 24.8%), environmental information processing (2455, 8.6%), and organismal systems (6273, 21.9%). Notably, sensory system (264, 1.97%) and environmental adaptation (96, 0.72%), which were closely associated with activities, were represented. Several unigenes belonged to phototransduction, olfactory transduction, taste transduction, and circadian rhythm-fly pathways (Supplementary Table S5).

**Figure 5:**
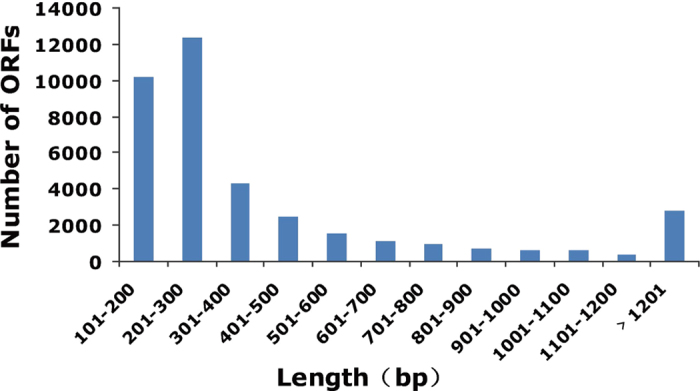
Length distribution of identified ORFs.

Sequence orientations were determined according to the best hit in the database. For protein coding region prediction, All-unigenes were aligned by blast search against the nr, Swiss-Prot, KEGG, and COG databases, using an E-value cut-off of 10^−5^. A total of 21,166 coding sequences (CDSs) mapped to the protein database, and there were 1591 predicted CDSs (ESTscan-CDSs) (Supplementary Fig. S1). Among All-unigenes with CDSs, 13,292 (62.8%) were <500 bp, while 7260 (34.3%) were 500–2000 bp and 144 were >3000 bp. We performed open reading frame (ORF) predictions using getorf software from the EMBOSS (v. 6.0.1) analysis package, and detected 38,580 ORFs in 46,327 All-unigenes. The majority of ORFs (70.04%) contained 101–400 bp, whereas 11,997 ORFs (19.82%) contained 401–1000 bp and 3912 ORFs (10.14%) exceeded 1,000 bp (Fig. 5).

### Possible genes related to phototransduction-fly and circadian rhythm-fly pathways

We investigated the presence of phototransduction and circadian rhythm pathways in the *M. separata* transcriptome and mapped the transcripts to pathways in the KEGG database (Supplementary Figure S2). The putative identified genes required for phototransduction-fly, such as *Gq, PLCβ, PKC, TRP, TRPL, INAD, arr2, NINAC* and *CamkII*, and those for circadian rhythm, such as *Dbt, Per, Tim, Sgg, Cyc, dCLK, Vri* and *Pdp*, respectively, were first identified in *M. separata* (Supplementary Tables S6 and S7). *TRP, Arr2, PLCβ, Dbt, Per, Tim*, and *Vri* had full ORFs. We identified three opsins, UV wavelength-sensitive opsin (Unigene9668_All), long-wavelength opsin (Unigene13996_All), and blue-sensitive visual pigment (CL2771.Contig1_All, CL2771.Contig2_All, CL2771.Contig4_All and CL2771.Contig5_All), all with amino acids homologies of 100% with KF539458, KF539446, and KF539428, respectively^45^. Meanwhile, a putative new opsin (Unigene10271_All) with an amino acid homology >76% with the predicted UV-sensitive-like opsin from *Amyelois transitella* was also identified. In this study, we detected *TRP* (Unigene9544_All) and *TRP-like* (*TRPL*) (CL3715.Contig1_All), which both had high amino acid homologies with genes in *Papilio xuthus*. However, *DAGL* (*DAG* lipase), which was shown to be necessary for transient receptor potential (TRP) channel regulation in *Drosophila* photoreceptors, was not identified in this species.

### Interspecific comparisons

The distribution patterns of *M. separata* All-unigenes were analysed across two other representative noctuid insects, *Helicoverpa armigera* and *Helicoverpa assulta*. Totals of 1463 (3.16%) and 1393 (3.00%) *M. separata* unigenes were similar in *H. armigera* and *H. assulta* transcripts, respectively (Supplementary File 2). Similarly, 591 (1.28%) and 1167 (2.52%) unigenes were unique to *H. armigera* and *H. assulta,* respectively (Supplementary File 2).

### Identification of DEGs

**Figure 6:**
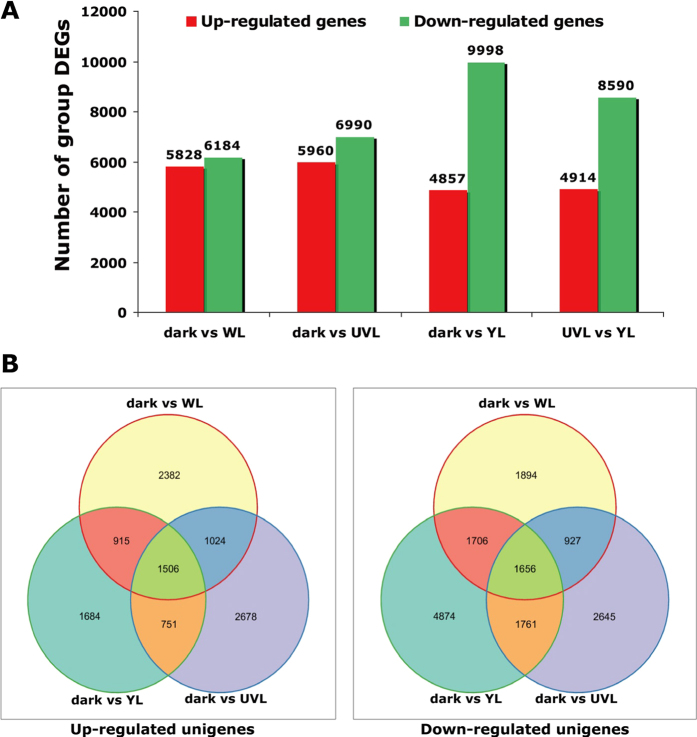
(**A**)Genes differentially expressed between groups. Differentially expressed genes were identified based on a ≥2-fold change and FDR ≤ 0.001. (**B**) Venn diagrams showing overlap of DEGs in response to three light stresses. The number of unigenes in each region of the diagrams are indicated. The Venn diagrams depict the overlaps between each pairwise comparison. Left-right: Venn maps of up-regulated (left) and down-regulated unigenes (right).

To gain a global view of transcript expression in *M. separata* in response to light stress, we compared the DEGs in different samples using SOAP (version 2.21). DEGs were selected by the fragment per kilobase of exon model per million mapped reads (FPKM) method with conditions of FDR (false discovery rate) ≤0.001 and the absolute value of log_2_Ratio ≥1. All the DEGs are presented in Fig. 6 and Supplementary File 3. For dark vs WL, there were 5828 and 6184 up- and down-regulated genes, respectively; for dark vs UVL, there were 5960 and 6990 up- and down-regulated genes, respectively; for dark vs YL, there were 4857 and 9998 up- and down-regulated genes, respectively; and for UVL vs YL, there were 4914 and 8590 up- and down-regulated genes, respectively. Totals of 2382 and 1894 DEGs were uniquely up- and down-regulated, respectively in the WL-exposed group, 2678 were up-regulated and 2645 were down-regulated in the UVL group, and 1684 were up-regulated and 4874 were down-regulated in the YL group. For all pairwise comparisons, many DEGs were specific for each light-stress treatment (Supplementary File 3).

All significantly induced and repressed genes derived from pairwise comparisons were compared with each other, and the overlaps between each pairwise comparison are depicted in Fig. 6B. These results suggest that genes are differentially expressed in response to light exposure in *M. separata*, particularly under conditions of light stress.

### Annotation analysis of DEGs

To clarify the functions of DEGs involved in response to light treatment, we mapped all the DEGs to terms in the GO database and compared the results with the whole transcriptome background. The DEGs had a GO ID and could be categorized into small functional groups in three main GO categories of biological process, cellular component, and molecular function. Based on sequence homology, we identified significant DEGs annotated by the GO database. We categorised 53, 52, 54, and 52 functional groups in dark-vs-WL, dark-vs-UVL, dark-vs-YL, and UVL-vs-YL, respectively (Supplementary File 4, Fig. S3). Among these groups, “cellular process” and “single-organism process” were dominant within the “biological process” category, “cell” and “cell part” categories were dominant in the “cellular component” category, and “binding” and “catalytic activity” were dominant in the “molecular function” category.

We investigated the biochemical pathways of these DEGs further by mapping all the DEGs to terms in the KEGG database and comparing this with the whole transcriptome background. The DEGs had a KO ID and could be categorized into small pathways (Supplementary File 5, Fig. S4). Of the 12,012, 12,950, and 14,855 DEGs between the dark-vs-WL, dark-vs-UVL, and dark-vs-YL groups, 1938, 2482, and 2925 unigenes had KO IDs and could be categorized into 247, 248, and 252 pathways, respectively. Genes involved in metabolic pathways were the most significantly enriched.

### Validation of expression profiles by RT-qPCR

**Figure 7:**
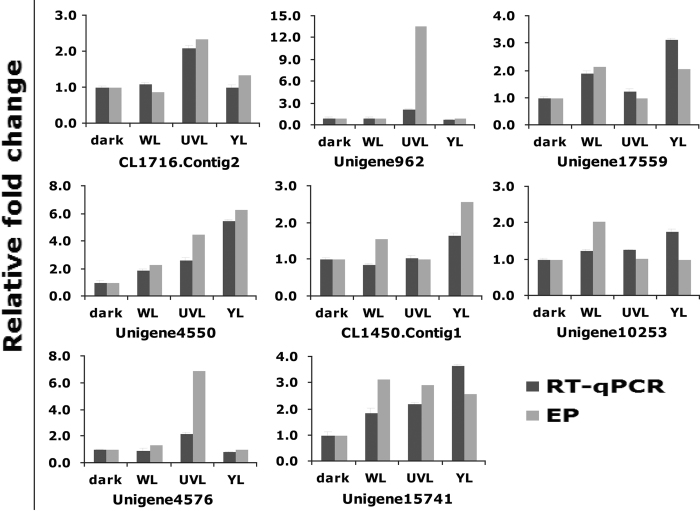
Comparison between RT-qPCR and expression profile data. Eight genes were analyzed by RT-qPCR and compared with expression profiles (EP). Data are presented as mean fold change ± standard error.

To confirm the expression profile data, we further examined the relative expression levels of eight selected genes and quantified the relative expression levels between dark and three light-treated samples by RT-qPCR. Three heads for each sample were used for each gene validation. Among the eight analyzed genes, seven were detected to have similar fold changes to the expression profile data (Fig. 7). These comparative analyses revealed a concordance rate >87.0% between RT-qPCR analysis and expression profile data, thus validating the accuracy and reliability of the sequencing data.

## Discussion

Light is an important environmental signal for most insects^19^,^27^,^46^. Light stress refers to the detrimental effect that exposure to insufficient or excess levels of light can have on an organism’s function and development. Mortality of second instar larvae of *M. separata* was shown to be higher than in the third and fourth instar larvae after UV-A radiation^34^, and increased UV-A radiation of pupae could decrease the rate of adult emergence in this species^34^. As a major abiotic force, light has a wide range of biological effects on insects^19^,^24^,^47^, which are known to be highly sensitive to artificial light^23^. Light can influence the physiology, behaviour, and reproduction of insects, especially nocturnal insects^22^,^23^,^24^,^25^, and these influences may vary depending on the wavelength of the light^19^,^27^. Numerous studies have discussed the ecological impacts of light on insects, including disturbances of biological rhythms, orientation, and migration, and of basal activities such as foraging and mating behaviours, as well as effects on reproductive success^18^,^24^,^27^. However, the effects of light on nocturnal insects at the molecular level are largely unknown because of a lack of genomic information.

*M. separata* is an important nocturnal pest. Although full-genome information is lacking for *M. separata*, our collection of unique transcripts may represent a significant proportion of the functional genes in this species. In this study, we used the heads of *M. separata* exposed to different environmental light conditions to perform a genome-wide investigation by transcriptional sequencing and gene expression profile analysis. The results of this study provide the first transcriptomic analysis of *M. separata* in relation to light stress, and also provide useful information for studying the molecular mechanisms of responses to light stress in nocturnal insects.

In this study, we assembled 46,327 unigenes from the heads of the nocturnal moth *M. separata*, with or without light exposure. Among our transcriptome data, 21,328 unigenes were annotated and showed specific Swiss-Prot matches. The annotated unigenes were further used for functional annotation and classification using the GO and KEGG databases, which provide information about probable biological functions and biosynthesis pathways. Functional annotation of these unigenes demonstrated that they covered most biological processes. Of the unigenes with significant hits in the nr database, 10,621 were categorized into 57 sub-categories, and 13,394 unigenes were mapped into 258 significant KEGG pathways. Regarding secondary metabolite pathways, genes for sensory system, environmental adaptation, and digestive system were strongly represented. These categories were considered to be major components of the response to environmental light stress. However, up to 47.62% of unigenes were not annotated by BLAST, possibly because of limited information about the genomes or transcriptomes of the moth and its related species^48^,^49^. These unmatched unigenes might be candidates for novel gene discoveries.

We investigated 88, 68, 52, 72, and 36 unigenes involved in phototransduction-fly, phototransduction, circadian rhythm-fly, olfactory transduction, and taste transduction, respectively, by transcriptomic analysis. Many genes involved in sensory system and environmental adaptation were found to undergo expression changes through comparative transcriptomic analyses. Phototransduction and circadian rhythm are both regulated by light and represent important biological processes in living organisms. TRP channels have important functions in phototransduction in adult insect visual systems^50^. These channels can detect and respond to light changes in the environment and thereby influence many insect behaviours^51^. Previous studies have indicated that many important proteins are involved in phototransduction and circadian rhythms. However, our current understanding of insect phototransduction and circadian rhythm has mainly been derived from *D. melanogaster*^36^,^39^. Rhodopsin, G(alpha)q, PLC, PKC, TRP, and TRPL are important transduction proteins in phototransduction processes, and genes encoding these proteins were identified in *M. separata* in this study. Several well-known *Drosophila* circadian clock genes, such as *period, timeless, Clock, vrille* and *Pdp1,* have been also found in this species. Opsins are membrane proteins, and play key roles in insect phototransduction by absorbing light and activating G-protein^52^. In addition to the three kinds of opsins previously identified in *M. separata*^45^, we also found a putative new opsin. The unigenes required for phototransduction-fly and circadian rhythm identified in *M. separata* showed shared descent with those of *D. melanogaster* and other Lepidoptera. *INAD* and *NINAC* were first identified in Lepidoptera in this study. These findings have thus greatly enriched our current knowledge of *M. separata* gene expression profiles, and provided molecular bases for further studies of the response mechanisms of *M. separata* to light stress.

The current transcriptomic analysis identified many DEGs between insects treated with different wavelengths of light, with different numbers of DEGs in GO and KEGG terms and pathways. The regulated unigenes covered a variety of functions, and analysis of GO categories revealed enrichment of genes involved in stress response, circadian rhythm, phototransduction, olfactory transduction, taste transduction, and DNA repair. Many genes were differentially up-regulated or down-regulated in light-treated compared with dark-treated *M. separata*. Comparison between the UVL and YL libraries identified 4,914 up-regulated and 8590 down-regulated unigenes. Compared with the dark-treated group, 15 genes encoding heat shock proteins were up-regulated or down-regulated, while genes such as cytochrome P450 (Unigene13937_All) was repressed in WL, YL and UVL groups. Antennal binding protein (Unigene4550_All) was up-regulated in the UVL and YL but down-regulated in the WL group in the olfactory transduction pathway. Overall, more genes were down-regulated than up-regulated in the three treated groups compared with the dark control, suggesting that light largely inhibited gene expression, with possible implications for impaired biological functioning. However, further studies are needed to determine the significance of the altered expression of specific genes or pathways.

In summary, we obtained 46,327 unigenes from *M. separata* by transcriptomic sequencing, and identified numerous light stress-associated DEGs and signal pathways by gene expression profiling. These data suggest that the moth might respond to light stress at the transcriptomic level by decreasing or increasing expression levels of genes related to metabolism, phototransduction, olfactory transduction, and taste transduction. Overall, these results provide the first informative reference dataset for future studies on global and specific responses of insects to light at the molecular level, and will facilitate further gene discoveries and biomarker identification in *M. separata* and other nocturnal insects. Our work may also provide some new insights into the development of light traps for forecasting the outbreaks and controlling the activities of *M. separata*.

## Materials and Methods

### Insect rearing and light treatments

*M. separata* larvae were fed on maize seedlings in the laboratory at 25 ± 1 °C and 70% ± 5% relative humidity, with a photoperiod of 14 h:10 h (light:dark). Pupae were sexed and kept separately until eclosion. *M. separata* used in all experiments were obtained from a colony maintained at the Institute of Plant Protection, Henan Academy of Agricultural Sciences, Zhengzhou, China. Adult moths were fed on a 10% honey solution.

Three-day-old adult moths (female:male 1:1) were collected at 06:00 and then treated with white light (400–750 nm and 1500 lux) for 6 h. The moths were then divided randomly into a group (WL) maintained under white light, while the other moths were further divided into three groups: dark, moths maintained in complete darkness for 3 h after constant white light for 6 h; UVL, moths maintained under UV light (365 nm, 110 lux) for 3 h after constant white light for 6 h and dark for 3 h; YL, moths maintained under yellow light (589 nm, 110 lux) for 3 h after 6 h white light and 3 h dark treatment. Experiments were performed a total of three times. For each treatment, at least 60 adult heads were used and the collected samples were frozen immediately in liquid nitrogen after sampling and stored at −80 °C until further use.

### RNA extraction, cDNA library construction, and Illumina sequencing

Total RNA was extracted using TRIzol reagent (Invitrogen Carlsbad, CA, USA) according to the manufacturer’s instructions. For transcriptome sequencing, equal amounts of total RNA from four samples were pooled and treated with DNase. The concentration and quality of DNase-treated RNA was evaluated using a NanoDrop 2000 UV–vis Spectrophotometer (Thermo Scientific, Waltham, MA, USA) and 1% formaldehyde gel electrophoresis. RNA integrity was assessed on an Agilent 2100 Bioanalyzer (Agilent Technologies, Inc., CA, USA). mRNA was isolated from total RNA using magnetic beads with oligo(dT) and sheared into short fragments using fragmentation buffer. cDNA was then synthesized from the mRNA fragments using SuperScript III Reverse Transcriptase (Invitrogen), according to the manufacturer’s manual. The short fragments were then connected with sequencing adapters and analyzed by agarose gel electrophoresis. Suitable fragments were enriched by PCR amplification to construct the cDNA library. Four pooled cDNA libraries were constructed using an mRNA-Seq assay for paired-end transcriptome sequencing and sequenced on Illumina HiSeq2000 system at Beijing Genomics Institute (BGI, Shenzhen, China) to generate 100 base single-end reads. The raw data from Illumina deep-sequencing have been deposited in the Sequence Read Archive (NCBI) (accession numbers: SRR3658912–SRR3658915).

### Functional annotations and ORF prediction of All-unigenes

The raw reads generated by Hiseq 2000 were filtered to remove low-quality reads (reads containing adaptor, reads containing >5% unknown nt “N”, and reads with >20% quality value ≤10). Clean reads were then *de novo* assembled using Trinity software (version release-20130225) to generate a collection of non-redundant unigenes without a reference genome^53^. In this study, Trinity generated transcriptome assemblies from short-read sequences using the de Bruijn graph algorithm, and K-mer was set at 25 bp. Trinity first combined clean reads to form longer contigs, and then assembled contigs into unigenes. Finally, unigenes from each sample’s assembly were further processed by sequence splicing and redundancy removal with sequence-clustering software to acquire non-redundant unigenes that were as long as possible, to form global transcriptome data for *M. separata* heads. After clustering, the unigenes were divided into clusters and singletons.

Assembled unigenes were subjected to blastx (BLAST, the basic local alignment search tool) alignment (E value < 1e-5) and several protein databases, including nt, Swiss-Prot, KEGG, COG, and Interpro^54^,^55^. We performed GO functional classification for All-unigenes using Blast2GO, according to molecular function, biological process, and cellular component^56^,^57^. The genes’ complex biological behaviours were further examined by pathway annotation using KEGG identifiers (http://www.genome.jp/kegg/). These blasted unigenes were then used to extract CDS and translated into peptide sequences. Unigenes without BLAST hits were predicted using ESTScan (version v3.0.2) and then translated into peptide sequences^58^. The ORFs of unigenes were predicted using getorf software (http://www.bioinformatics.nl/cgi-bin/emboss/getorf)^59^. For a coding sequence with more than two ORFs, the longest one was identified as the sequence of interest.

### Comparative transcriptomics

The transcriptome datasets of *H. armigera* (SRX518090) and *H. assulta* (SRX733617 and SRX707455) were downloaded from the NCBI Sequence Read Archive and *de novo* assembled using Trinity (version release-20130225). All-unigenes of *M. separata* were mapped to the transcriptomes of *H. armigera* and *H. assulta*. The BLASTn algorithm was used to test sequence similarities, with a threshold E-value < 1e^−5^. The sequences were compared with the longest contig from each of the transcripts identified in the *M. separata* transcriptome.

### Identification of DEGs

DEGs were determined based on their expression abundances in the different treatment groups using SOAP (version 2.21) (http://soap.genomics.org.cn/soapaligner.html). FPKM values were used to evaluate expression abundances of genes and to quantify transcript levels among the different samples^59^. DEGs were based on differences in expression abundances among the four cDNA libraries, with FDR ≤ 0.001 and the absolute value of log_2_Ratio ≥ 1 as the threshold for significant differential gene expression^60^. DEGs were also annotated using the GO database^57^, and the numbers of DEGs in each GO term were calculated. KEGG pathway analysis of the DEGs was also performed to identify the associated biochemical and signal transduction pathways^54^.

### RT-PCR validation

The expression patterns of selected genes were analyzed by RT-qPCR using a Thermal Cycler Dice Real Time System Lite (TaKaRa, Japan). Each reaction contained 12.5 μl of 2 × SYBR Premix Ex Taq II (TaKaRa), 2 μl of cDNA, and 1 μM of gene-specific primers in a final volume of 25 μl. The PCRs were performed under the following conditions: 95 °C for 5 min, followed by 40 cycles of 95 °C for 5 s, 60 °C for 30 s and 72 °C for 20 s. Relative expression levels of each gene were calculated using the 2^−ΔΔCt^ algorithm^61^ by normalizing to expression of *M. separata* arginine kinase gene, which was used as an internal control. Three technical replicates were used for each sample and the data were shown as means ± standard errors. The primer sequences are listed in Supplementary Table S8.

## Additional Information

**How to cite this article:** Duan, Y. *et al*. Transcriptome analysis of molecular mechanisms responsible for light-stress response in *Mythimna separata* (Walker). *Sci. Rep.* **7**, 45188; doi: 10.1038/srep45188 (2017).

**Publisher's note:** Springer Nature remains neutral with regard to jurisdictional claims in published maps and institutional affiliations.

## Supplementary Material

Supplementary Information

Supplementary Dataset 1

Supplementary Dataset 2

Supplementary Dataset 3

Supplementary Dataset 4

Supplementary Dataset 5

## Figures and Tables

**Table 1:** Summary of transcriptome sequencing.

	dark	White light	UV light	Yellow light
Raw Reads	58,194,634	61,409,338	59,133,346	56,299,308
Clean Reads (n)	54,402,458	55,437,108	50,414,324	52,197,036
Base number (bp)	4,896,221,220	4,989,339,720	4,537,289,160	4,697,733,240
Q20 percentage (%)	98.12	97.04	96.93	98.23
N percentage (%)	0.00	0.00	0.04	0.00
GC percentage (%)	48.33	47.57	47.31	48.78

**Table 2:** Unigenes assembled using the Trinity method.

	Total number (percentage)				
	dark	White light	UV light	Yellow light	All unigenes
300–500 bp	22,730 (67.38%)	24,166 (73.18%)	25,757 (74.05%)	16,501 (74.90%)	32,421 (69.50%)
600–1000 bp	6688 (19.82%)	5463 (16.54%)	5777 (16.61%)	3632 (16.49%)	8025 (17.3%)
1100–2000 bp	3164 (9.38%)	2616 (7.92%)	2449 (7.04%)	1558 (7.07%)	4076 (8.80%)
2100–3000 bp	797 (2.36%)	553 (1.67%)	545 (1.57%)	240 (1.09%)	1226 (2.60%)
≥3000 bp	357 (1.06%)	223 (0.68%)	257 (0.74%)	99 (0.50%)	579 (1.25%)
Total number of clusters	5870	5266	5432	2804	11130
Total number of singletons	27,866	27,755	29,353	19,266	35,197
Total number of unigenes	33,736	33,021	34,785	22,030	46,327
Exclusive	6916	6175	6935	2563	—
Total length	19,307,242	16,828,834	17,423,358	10,453,549	26,456,921
N50 length	750	620	595	550	795
Mean length	571.85	509.64	500.89	474.51	571
