# Supplementary material for: Transcriptome analysis of molecular mechanisms responsible for light-stress response in Mythimna separata (Walker)
Source: Sci Rep. 2017 Mar 27;7:45188. doi: 10.1038/srep45188 (PMC5367045; doi:10.1038/srep45188)
Supplement: Supplementary Information [file srep45188-s1.pdf]

Transcriptome analysis of molecular mechanisms responsible for light-stress response in *Mythimna separata* (Walker)

Yun Duan, ZhongJun Gong, RenHai Wu, Jin Miao, YueLi Jiang, Tong Li, XiaoBo Wu, YuQing Wu*

Institute of Plant Protection, Henan Academy of Agricultural Sciences, Key Laboratory of Crop Pest Control of Henan Province, Key Laboratory of Integrated Pest Management on Crops in Southern Region of North China, Zhengzhou 450002, China.

*Corresponding author: Y.Q. Wu. (email: yuqingwu36@hotmail.com)

Supplementary information

Table S1. Summary of GO assignments of *Mythimna separata* transcriptome.

Ontology	Class	Number of genes	Percent
molecular_function	antioxidant activity	38	0.441%
molecular_function	binding	5475	6.360%
molecular_function	catalytic activity	4897	5.690%
molecular_function	channel regulator activity	19	0.0220%
molecular_function	electron carrier activity	81	0.094%
molecular_function	enzyme regulator activity	364	0.423%
molecular_function	metallochaperone activity	5	0.0058%
molecular_function	molecular transducer activity	445	0.517%
molecular_function	Morphogen activity	2	0.00232%
molecular_function	nucleic acid binding transcription factor activity	356	0.414%
molecular_function	nutrient reservoir activity	2	0.00232%
molecular_function	protein binding transcription factor activity	108	0.126%
molecular_function	protein tag	1	0.00116%
molecular_function	receptor activity	364	0.423%
molecular function	receptor regulator activity	2	0.00232%
molecular_function	structural molecule activity	378	0.439%
molecular_function	translation regulator activity	11	0.0128%
molecular_function	transporter activity	948	1.101%
cellular_component	cell	5220	6.065%
cellular_component	cell junction	322	0.374%
cellular_component	cell part	5220	6.065%
cellular_component	extracellular matrix	86	0.100%
cellular_component	extracellular matrix part	30	0.0349%
cellular_component	extracellular region	337	0.392%
cellular_component	extracellular region part	163	0.189%
cellular_component	macromolecular complex	2197	2.553%
cellular_component	membrane	2514	2.921%
cellular_component	membrane part	1711	1.988%
cellular_component	membrane-enclosed lumen	684	0.795%
cellular_component	nucleoid	12	0.0139%
cellular_component	organelle	3554	4.129%
cellular_component	organelle part	2107	2.448%
cellular_component	synapse	392	0.4554%
cellular_component	synapse part	283	0.3288%
biological_process	biological adhesion	332	0.3857%
biological_process	biological regulation	3442	3.999%
biological_process	cellular process	6929	8.050%

Ontology	Class	Number of genes	Percent
biological_process	cell killing	5	0.00581%
biological_process	cellular component organization or biogenesis	2423	2.815%
biological_process	developmental process	2919	3.391%
biological_process	Establishment of localization	2036	2.365%
biological_process	growth	483	0.5612%
biological_process	immune system process	374	0.4345%
biological_process	localization	2363	2.745%
biological_process	locomotion	668	0.7761%
biological_process	metabolic process	5183	6.0216%
biological_process	multi-organism process	403	0.4682%
biological_process	multicellular organismal process	3127	3.633%
biological_process	negative regulation of biological process	910	1.057%
biological_process	Positive regulation of biological process	825	0.9585%
biological_process	regulation of biological process	3150	3.6597%
biological_process	reproduction	1008	1.1711%
biological_process	reproductive process	931	1.0816%
biological_process	response to stimulus	2693	3.1287%
biological_process	rhythmic process	125	0.1452%
biological_process	signaling	1934	2.2469%
biological_process	single-organism process	5482	6.369%

Table S2. Summary of COG classfication of *Mythimna separata* transcriptome.

Functional Code	Functional categories	Number of genes	Percent
A	RNA processing and modification	79	1.23%
B	Chromatin structure and dynamics	175	2.72%
C	Energy production and conversion	413	6.40%
D	Cell cycle control, cell division, chromosome partitioning	758	11.76%
E	Amino acid transport and metabolism	562	8.72%
F	Nucleotide transport and metabolism	152	2.36%
G	Carbohydrate transport and metabolism	797	12.37%
H	Coenzyme transport and metabolism	194	3.01%
I	Lipid transport and metabolism	411	6.38%
J	Translation, ribosomal structure and biogenesis	1329	20.62%
K	Transcription	991	15.38%
L	Replication, recombination and repair	1035	16.06%
M	Cell wall/membrane/envelope biogenesis	834	12.94%
N	Cell motility	263	4.08%
O	Posttranslational modification, protein turnover, chaperones	901	13.98%
P	Inorganic ion transport and metabolism	425	6.60%
Q	Secondary metabolites biosynthesis, transport and catabolism	368	5.71%
R	General function prediction only	2454	38.08%
S	Function unknown	984	15.28%
T	Signal transduction mechanisms	648	10.06%
U	Intracellular trafficking, secretion, and vesicular transport	552	8.57%
V	Defense mechanisms	153	2.37%
W	Extracellular structures	20	0.31%
Y	Nuclear structure	6	0.09%
Z	Cytoskeleton	274	4.25%

Table S3. Summary of KEGG annotations of *Mythimna separata* transcriptome.

Pathway	Pathway ID	Number of genes	Percent
Metabolic pathways	ko01100	1827	13.64%
Spliceosome	ko03040	491	3.67%
RNA transport	ko03013	470	3.51%
Focal adhesion	ko04510	449	3.35%
Regulation of actin cytoskeleton	ko04810	445	3.32%
Pathways in cancer	ko05200	411	3.07%
Epstein-Barr virus infection	ko05169	396	2.96%
Purine metabolism	ko00230	379	2.83%
Endocytosis	ko04144	364	2.72%
Amoebiasis	ko05146	363	2.71%
MAPK signaling pathway	ko04010	360	2.69%
mRNA surveillance pathway	ko03015	336	2.51%
Protein processing in endoplasmic reticulum	ko04141	317	2.37%
HTLV-I infection	ko05166	312	2.33%
Huntington's disease	ko05016	301	2.25%
Vibrio cholerae infection	ko05110	295	2.2%
Bile secretion	ko04976	287	2.14%
Alzheimer's disease	ko05010	282	2.11%
Tight junction	ko04530	281	2.1%
Vascular smooth muscle contraction	ko04270	275	2.05%
Phagosome	ko04145	262	1.96%
Pyrimidine metabolism	ko00240	254	1.9%
Ubiquitin mediated proteolysis	ko04120	253	1.89%
Herpes simplex infection	ko05168	252	1.88%
Insulin signaling pathway	ko04910	251	1.87%
Influenza A	ko05164	246	1.84%
Dilated cardiomyopathy	ko05414	244	1.82%
Transcriptional misregulation in cancer	ko05202	242	1.81%
Hypertrophic cardiomyopathy (HCM)	ko05410	234	1.75%
Lysosome	ko04142	229	1.71%
Calcium signaling pathway	ko04020	228	1.70%
Adherens junction	ko04520	228	1.70%
Chemokine signaling pathway	ko04062	223	1.66%
ECM-receptor interaction	ko04512	222	1.66%
ABC transporters	ko02010	201	1.50%
Salmonella infection	ko05132	201	1.50%
Salivary secretion	ko04970	200	1.49%
Wnt signaling pathway	ko04310	200	1.49%
Neuroactive ligand-receptor interaction	ko04080	198	1.48%
Fc gamma R-mediated phagocytosis	ko04666	197	1.47%

Pathway	Pathway ID	Number of genes	Percent
RNA degradation	ko03018	197	1.47%
Tuberculosis	ko05152	195	1.46%
Toxoplasmosis	ko05145	189	1.41%
Axon guidance	ko04360	187	1.40%
Pancreatic secretion	ko04972	187	1.40%
Pathogenic Escherichia coli infection	ko05130	176	1.31%
Peroxisome	ko04146	174	1.3%
RNA polymerase	ko03020	171	1.28%
Ribosome biogenesis in eukaryotes	ko03008	169	1.26%
Leukocyte transendothelial migration	ko04670	168	1.25%
Oxidative phosphorylation	ko00190	168	1.25%
Bacterial invasion of epithelial cells	ko05100	163	1.22%
Protein digestion and absorption	ko04974	163	1.22%
Measles	ko05162	162	1.21%
Cell cycle	ko04110	157	1.17%
Neurotrophin signaling pathway	ko04722	156	1.16%
Prion diseases	ko05020	153	1.14%
Glycerolipid metabolism	ko00561	147	1.10%
Parkinson's disease	ko05012	145	1.08%
Dopaminergic synapse	Ko04728	145	1.08%
Synaptic vesicle cycle	ko04721	144	1.08%
Glycerophospholipid metabolism	ko00564	144	1.08%
Starch and sucrose metabolism	ko00500	144	1.08%
Shigellosis	ko05131	144	1.08%
Amyotrophic lateral sclerosis (ALS)	ko05014	143	1.07%
PPAR signaling pathway	ko03320	141	1.05%
Lysine degradation	ko00310	141	1.05%
Gastric acid secretion	ko04971	141	1.05%
T cell receptor signaling pathway	ko04660	140	1.05%
Drug metabolism - other enzymes	ko00983	139	1.04%
Cardiac muscle contraction	ko04260	137	1.02%
GnRH signaling pathway	ko04912	135	1.01%
Alcoholism	ko05034	135	1.01%
Cell adhesion molecules (CAMs)	ko04514	134	1.00%
Melanogenesis	ko04916	132	0.99%
Small cell lung cancer	ko05222	131	0.98%
Phosphatidylinositol signaling system	ko04070	130	0.97%
Pentose and glucuronate interconversions	ko00040	126	0.94%
Oocyte meiosis	ko04114	126	0.94%
Legionellosis	ko05134	125	0.93%
Glutamatergic synapse	ko04724	125	0.93%
Dorso-ventral axis formation	ko04320	124	0.93%

Pathway	Pathway ID	Number of genes	Percent
Progesterone-mediated oocyte maturation	ko04914	124	0.93%
Prostate cancer	ko05215	123	0.92%
Galactose metabolism	ko00052	120	0.9%
Viral myocarditis	ko05416	115	0.86%
Cholinergic synapse	ko04725	113	0.84%
Vasopressin-regulated water reabsorption	ko04962	112	0.84%
Gap junction	ko04540	109	0.81%
Long-term potentiation	ko04720	109	0.81%
Metabolism of xenobiotics by cytochrome P450	ko00980	108	0.81%
Amino sugar and nucleotide sugar metabolism	ko00520	108	0.81%
ErbB signaling pathway	ko04012	108	0.81%
Serotonergic synapse	ko04726	108	0.81%
Epithelial cell signaling in Helicobacter pylori infection	ko05120	108	0.81%
Fat digestion and absorption	ko04975	108	0.81%
Carbohydrate digestion and absorption	Ko04973	105	0.78%
Retinol metabolism	ko00830	105	0.78%
Drug metabolism - cytochrome P450	ko00982	105	0.78%
Antigen processing and presentation	ko04612	103	0.77%
Basal transcription factors	ko03022	102	0.76%
Cytosolic DNA-sensing pathway	ko04623	101	0.75%
GABAergic synapse	ko04727	100	0.75%
Arrhythmogenic right ventricular cardiomyopathy (ARVC)	ko05412	100	0.75%
Retrograde endocannabinoid signaling	ko04723	99	0.74%
Morphine addiction	ko05032	99	0.74%
Hepatitis C	ko05160	97	0.72%
Fructose and mannose metabolism	ko00051	97	0.72%
Ribosome	ko03010	97	0.72%
Inositol phosphate metabolism	ko00562	96	0.72%
Amphetamine addiction	ko05031	95	0.71%
Notch signaling pathway	ko04330	94	0.70%
Renal cell carcinoma	ko05211	93	0.69%
Adipocytokine signaling pathway	ko04920	91	0.68%
Mineral absorption	Ko04978	90	0.67%
Phototransduction - fly	ko04745	88	0.66%
Glioma	ko05214	88	0.66%
Pyruvate metabolism	ko00620	88	0.66%
Aminoacyl-tRNA biosynthesis	ko00970	87	0.65%
TGF-beta signaling pathway	ko04350	87	0.65%

Pathway	Pathway ID	Number of genes	Percent
Chagas disease (American trypanosomiasis)	ko05142	85	0.63%
VEGF signaling pathway	ko04370	85	0.63%
Glutathione metabolism	ko00480	85	0.63%
Valine, leucine and isoleucine degradation	ko00280	83	0.62%
mTOR signaling pathway	ko04150	82	0.61%
Arginine and proline metabolism	ko00330	82	0.61%
Fanconi anemia pathway	ko03460	81	0.60%
Glycine, serine and threonine metabolism	ko00260	80	0.60%
Hedgehog signaling pathway	ko04340	79	0.59%
Endometrial cancer	ko05213	78	0.58%
Complement and coagulation cascades	ko04610	77	0.57%
Glycolysis/Gluconeogenesis	ko00010	77	0.57%
Fatty acid metabolism	ko00071	76	0.57%
Jak-STAT signaling pathway	ko04630	75	0.56%
Steroid hormone biosynthesis	ko00140	74	0.55%
Type II diabetes mellitus	ko04930	74	0.55%
Malaria	ko05144	73	0.55%
Vitamin digestion and absorption	ko04977	73	0.55%
Tryptophan metabolism	ko00380	73	0.55%
Rheumatoid arthritis	ko05323	72	0.54%
Olfactory transduction	ko04740	72	0.54%
Tyrosine metabolism	ko00350	72	0.54%
p53 signaling pathway	ko04115	71	0.53%
Toll-like receptor signaling pathway	ko04620	70	0.52%
Basal cell carcinoma	ko05217	69	0.52%
NF-kappa B signaling pathway	ko04064	69	0.52%
Fc epsilon RI signaling pathway	ko04664	68	0.51%
Phototransduction	ko04744	68	0.51%
B cell receptor signaling pathway	ko04662	68	0.51%
Nucleotide excision repair	ko03420	67	0.50%
Osteoclast differentiation	ko04380	67	0.50%
Colorectal cancer	ko05210	67	0.50%
Chronic myeloid leukemia	ko05220	67	0.50%
Pancreatic cancer	ko05212	66	0.49%
Cocaine addiction	ko05030	65	0.49%
Endocrine and other factor-regulated calcium reabsorption	ko04961	64	0.48%
Hematopoietic cell lineage	ko04640	62	0.46%
Porphyrin and chlorophyll metabolism	ko00860	62	0.46%
Citrate cycle (TCA cycle)	ko00020	62	0.46%
Pertussis	ko05133	61	0.46%
Natural killer cell mediated cytotoxicity	ko04650	61	0.46%

Pathway	Pathway ID	Number of genes	Percent
Propanoate metabolism	ko00640	60	0.45%
Long-term depression	ko04730	59	0.44%
Other types of O-glycan biosynthesis	ko00514	59	0.44%
Phenylalanine metabolism	ko00360	59	0.44%
Ascorbate and aldarate metabolism	ko00053	58	0.43%
beta-Alanine metabolism	ko00410	57	0.43%
Insect hormone biosynthesis	ko00981	55	0.41%
MAPK signaling pathway - fly	ko04013	55	0.41%
N-Glycan biosynthesis	ko00510	55	0.41%
Apoptosis	ko04210	53	0.40%
Cysteine and methionine metabolism	ko00270	52	0.39%
Circadian rhythm - fly	ko04711	52	0.39%
Melanoma	ko05218	50	0.37%
Acute myeloid leukemia	ko05221	49	0.37%
Alanine, aspartate and glutamate metabolism	ko00250	49	0.37%
Nicotine addiction	ko05033	48	0.36%
alpha-Linolenic acid metabolism	ko00592	48	0.36%
Pentose phosphate pathway	ko00030	48	0.36%
Proteasome	ko03050	48	0.36%
Cytokine-cytokine receptor interaction	ko04060	48	0.36%
Glycosaminoglycan biosynthesis - heparan sulfate	ko00534	47	0.35%
Non-small cell lung cancer	ko05223	47	0.35%
Biosynthesis of unsaturated fatty acids	ko01040	47	0.35%
Collecting duct acid secretion	ko04966	46	0.34%
Ether lipid metabolism	ko00565	45	0.34%
Linoleic acid metabolism	ko00591	44	0.33%
Circadian rhythm - mammal	ko04710	44	0.33%
NOD-like receptor signaling pathway	ko04621	44	0.33%
Butanoate metabolism	ko00650	44	0.33%
Nicotinate and nicotinamide metabolism	ko00760	44	0.33%
Primary immunodeficiency	ko05340	43	0.32%
Sphingolipid metabolism	ko00600	43	0.32%
Glyoxylate and dicarboxylate metabolism	ko00630	41	0.31%
Staphylococcus aureus infection	ko05150	40	0.30%
Leishmaniasis	ko05140	40	0.30%
Fatty acid elongation	ko00062	40	0.30%
Thyroid cancer	ko05216	39	0.29%
Systemic lupus erythematosus	ko05322	38	0.28%
Homologous recombination	ko03440	38	0.28%
Histidine metabolism	ko00340	38	0.28%

Pathway	Pathway ID	Number of genes	Percent
Base excision repair	ko03410	36	0.27%
Steroid biosynthesis	ko00100	36	0.27%
DNA replication	ko03030	36	0.27%
Taste transduction	ko04742	36	0.27%
Ubiquinone and other terpenoid-quinone biosynthesis	ko00130	35	0.26%
Aldosterone-regulated sodium reabsorption	ko04960	35	0.26%
SNARE interactions in vesicular transport	ko04130	35	0.26%
Arachidonic acid metabolism	ko00590	34	0.25%
Renin-angiotensin system	ko04614	34	0.25%
Regulation of autophagy	ko04140	34	0.25%
Glycosylphosphatidylinositol(GPI)-anchor biosynthesis	ko00563	33	0.25%
Bladder cancer	ko05219	33	0.25%
One carbon pool by folate	ko00670	32	0.24%
RIG-I-like receptor signaling pathway	ko04622	31	0.23%
Other glycan degradation	ko00511	31	0.23%
Terpenoid backbone biosynthesis	ko00900	30	0.22%
Proximal tubule bicarbonate reclamation	ko04964	29	0.22%
Protein export	ko03060	28	0.21%
Mismatch repair	ko03430	27	0.20%
Folate biosynthesis	ko00790	26	0.19%
Glycosaminoglycan degradation	ko00531	21	0.16%
Pantothenate and CoA biosynthesis	ko00770	21	0.16%
Sulfur relay system	ko04122	20	0.15%
Autoimmune thyroid disease	ko05320	19	0.14%
Type I diabetes mellitus	ko04940	17	0.13%
Cyanoamino acid metabolism	ko00460	17	0.13%
Caffeine metabolism	ko00232	17	0.13%
Primary bile acid biosynthesis	ko00120	16	0.12%
Riboflavin metabolism	ko00740	16	0.12%
Mucin type O-Glycan biosynthesis	ko00512	15	0.11%
Fatty acid biosynthesis	ko00061	14	0.10%
African trypanosomiasis	ko05143	14	0.10%
Glycosphingolipid biosynthesis - ganglio series	ko00604	13	0.10%
Maturity onset diabetes of the young	ko04950	13	0.10%
Glycosaminoglycan biosynthesis - chondroitin sulfate	ko00532	12	0.09%
Synthesis and degradation of ketone bodies	ko00072	12	0.09%
Selenocompound metabolism	ko00450	11	0.08%
Sulfur metabolism	ko00920	11	0.08%

Pathway	Pathway ID	Number of genes	Percent
Glycosphingolipid biosynthesis -globo series	ko00603	11	0.08%
Glycosphingolipid biosynthesis -lacto and neolacto series	ko00601	10	0.07%
Glycosaminoglycan biosynthesis - keratan sulfate	ko00533	10	0.07%
Taurine and hypotaurine metabolism	ko00430	9	0.07%
Non-homologous end-joining	ko03450	9	0.07%
Valine, leucine and isoleucine biosynthesis	ko00290	8	0.06%
Lipoic acid metabolism	ko00785	6	0.04%
Butirosin and neomycin biosynthesis	ko00524	6	0.04%
D-Arginine and D-ornithine metabolism	ko00472	5	0.04%
Phenylalanine, tyrosine and tryptophan biosynthesis	ko00400	5	0.04%
Vitamin B6 metabolism	ko00750	5	0.04%
Biotin metabolism	ko00780	4	0.03%
Lysine biosynthesis	ko00300	3	0.02%
Asthma	ko05310	3	0.02%
Intestinal immune network for IgA production	ko04672	3	0.02%
D-Glutamine and D-glutamate metabolism	ko00471	2	0.01%
Allograft rejection	ko05330	2	0.01%
Graft-versus-host disease	ko05332	2	0.01%
Thiamine metabolism	ko00730	2	0.01%

Table S4. KEGG pathway classification of All-unigenes.

**Level 1**	**Level 2**	**All-unigenes with pathway annotation**
Metabolism	Biosynthesis of other secondary metabolites	23
	Metabolism of terpenoids and polyketides	85
	Glycan biosynthesis and metabolism	317
	Global map	1827
	Nucleotide metabolism	633
	Energy metabolism	179
	Lipid metabolism	820
	Carbohydrate metabolism	1169
	Amino acid metabolism	745
	Xenobiotics biodegradation and metabolism	352
	Metabolism of cofactors and vitamins	358
	Metabolism of other amino acids	186
Genetic information processing	Transcription	764
	Translation	1159
	Folding, sorting and degradation	898
	Replication and repair	294
Cellular processes	Cell communication	1067
	Cell motility	445
	Transport and catabolism	1063
	Cell growth and death	407
Human diseases	Cancers: Overview	653
	Infectious diseases: Viral	1465
	Infectious diseases: Parasitic	764
	Neurodegenerative diseases	288
	Infectious diseases: Bacterial	1508
	Cardiovascular diseases	693
	Substance dependence	442
	Cancers: Specific types	1000
	Endocrine and metabolic diseases	104
	Immune diseases	179
Environmental information processing	Signal transduction	1652
	Signaling molecules and interaction	602
	Membrane transport	201
Organismal systems	Digestive system	1354
	Circulatory system	412
	Endocrine system	908

	Immune system	1416
	Development	378
	Nervous system	1158
	Excretory system	286
	Sensory system	264
	Environmental adaptation	96

Table S5. Unigenes annotated to sensory system pathway and environmental adaptation pathway.

	Pathway	All-unigenes with pathway annotation	Pathway ID
Sensory system	Phototransduction-fly	88	Ko04745
	Phototransduction	68	Ko04744
	Olfactory transduction	72	ko04740
	Taste transduction	36	Ko04742
Environmental adaptation	Circadian rhythm-fly	52	Ko04711
	Circadian rhythm-mammal	44	Ko04710

Table S6. Genes required for phototransduction-fly identified in *Mythimna separata* transcriptome.

Gene symbol	Unigene ID	Length(bp)	Accession	Evalue	Species
TRP	Unigene2095_All	1885	EHJ65374.1	0	*D. plexippus*
TRPL	CL3715.Contig1_All	3924	EFA02859.1	0	*T. castaneum*
PKC	Unigene13203_All	2142	ABZ88709.1	0	*P. xylostella*
INAD	Unigene2186_All	3655	EFN61430.1	4E-122	*C. floridanus*
NINAC	Unigene124_All	4804	EGI60548.1	0	*A. echinatior*
Arr2	Unigene4315_All	1730	AEV53930.1	0	*M. vitrata*
IP3R	CL3091.Contig3_All	602	NP_730941.1	5E-57	*D. melanogaster*
Gq	CL2713.Contig2_All	3098	NP_001243986.1	1E-33	*B. mori*
PLCβ	Unigene2236_All	4375	XP_001653804.1	0	*A. aegypti*
RK	Unigene13152_All	2192	EFX84879.1	0	*D. pulex*
Camk2	Unigene5938_All	1878	NP_001177296.1	0	*B. mori*
rdgC	Unigene17017_All	2789	NP_788544.1	0	*D. melanogaster*
Uv-opsin	Unigene9668_All	2135	KF539458	0	*M. separata*
Long-opsin	Unigene13996_All	1851	KF539446	0	*M. separata*
Blue-pigment	CL2771.Contig1_All	2698	KF539428	0	*M. separata*

TRP: transient receptor potential channel; *TRPL*: transient receptor potential channel pyrexia-like; *PKC*: protein kinase C; *INAD*: inactivation-no-after-potential D protein; *NINAC*: inactivation nor after potential protein C; *Arr2*: arrestin 2; IP3R: inositol 1,4,5-trisphosphate receptor; *Gq*: G protein gamma subunit; *PLCβ*: phospholipase C beta; *RK*: G-protein-coupled receptor kinase; *rdgC*: retinal degeneration B; *Camk2*: Ca2+/calmodulin-dependent protein kinase II; Uv-opsin: Ultraviolet wavelength-sensitive opsin; Long-opsin: long-wavelength opsin; Blue-opsin: blue-sensitive visual pigment.

Table S7. Genes required for circadian rhythm-fly identified in *Mythimna separata* transcriptome.

Gene symbol	Unigene ID	Length(bp)	Accession	Evalue	Species
Dbt	Unigene13381_All	1387	AEJ38223.1	0	*S. exigua*
Per	CL163.Contig11_All	4662	ABF21088.1	0	*B. mori*
Tim	CL3181.Contig2_All	4807	AEJ38225.1	0	*S. exigua*
Sgg	CL1440.Contig1_All	1533	BU49716.1	0	*D. plexippus*
dCLK	CL3596.Contig2_All	1429	AAR14936.1	1E-111	*A. pernyi*
Cyc	CL2691.Contig2_All	1171	NP_001036982.1	1E-103	*B. mori*
Vri	Unigene2139_All	1469	AAS92609.1	8E-179	*A. pernyi*
Pdp	CL1044.Contig4_All	1655	NP_001261546.1	1E-89	*D. melanogaster*

Dbt: double-time; *Per*: period; Tim: timeless; *Sgg*: shaggy; *dCLK*: CLOCK; *Cyc*: cycle; *Vri*: vrille; *Pdp*: PAR-domain protein.

Table S8. Primers used for RT-qPCR in this study. F: forward primer sequence, R: reverse primer sequence, Length (bp): amplification length (base pair).

**Unigene ID**	**Primer sequence (5'-3')**	**Length (bp)**
AK	F: CAGTCTGGTGTTGAGAACTTGG	239
	R: TAGCCCTCCATGGAGCGTCC	
CL1716.Contig2_All	F:AGTGAAGACGACAGAGGTGGC	227
	R:GGTCAACAGCGTAAGTAGTGGC	
Unigene962_All	F: GGCGACGATTTGCTTGGT	104
	R: CCCAATCCCTCATCTTGTCTTC	
Unigene17559_All	F: GGTGGAGTGTTCGCAAGAAA	182
	R: TGGAGGAGAGGCAGAGGATG	
Unigene4550_All	F: GCTGGCGTGCTACATGAAGTG	221
	R:TGAGGAAGTAGTCAGCCTTGTTGG	
CL1450.Contig1_All	F:GCGGGCAATCAAAGAACC	101
	R:CCAATGGTATATTAATGCTGGATGG	
Unigene10253_All	F:AAAACGCCTAAATGCTTCATCC	191
	R:CTTTCACAGGGGCAGGTTTC	
Unigene4576_All	F:ACAACGGAGTCGGGAAGAAG	131
	R:TACTTGGCAGGGTATGGAGACA	
Unigene15741_All	F:CAAAGGCTGCTGTCGATAAATG	117
	R:CGAATATGAAGTCTGCTGGGTTG	

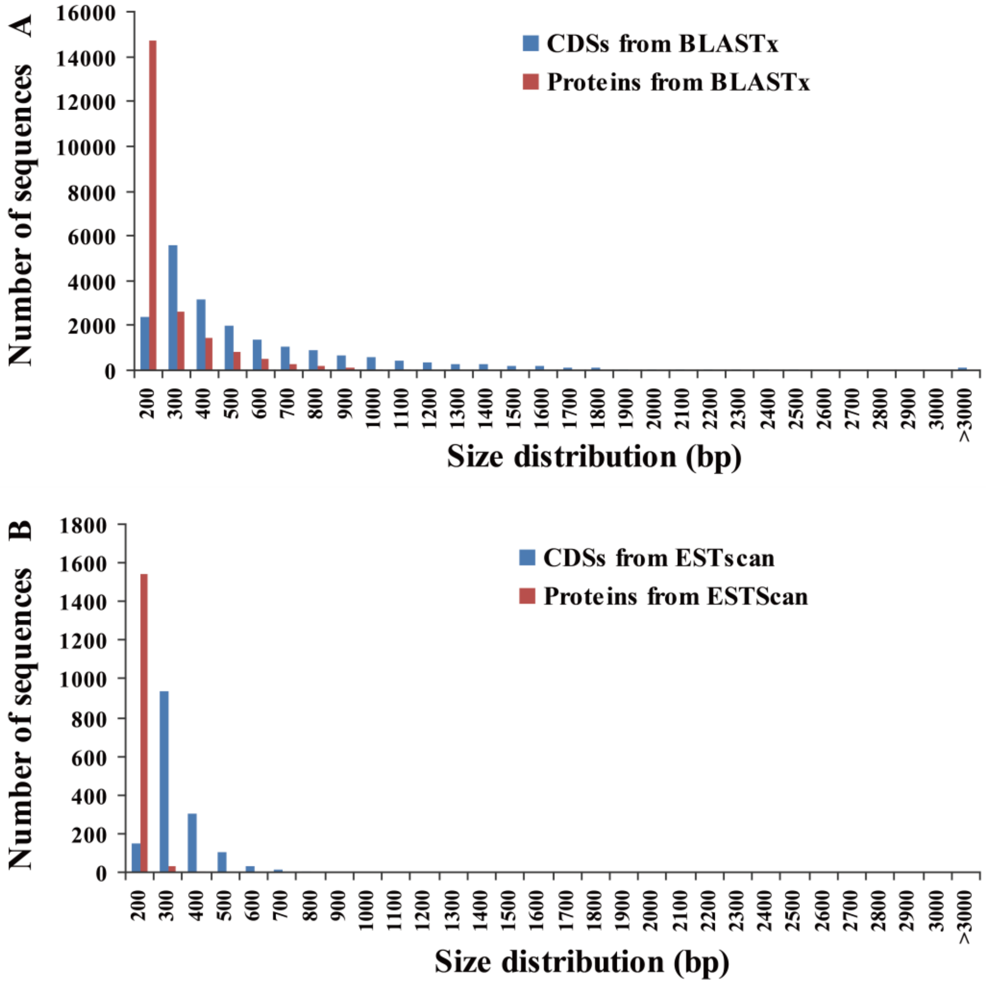

Figure S1. Coding sequence (CDS) of All-unigenes predicted by BLASTx and ESTScan. Size distribution of the CDSs and proteins based on BLASTx (A) and ESTScan (B).

A

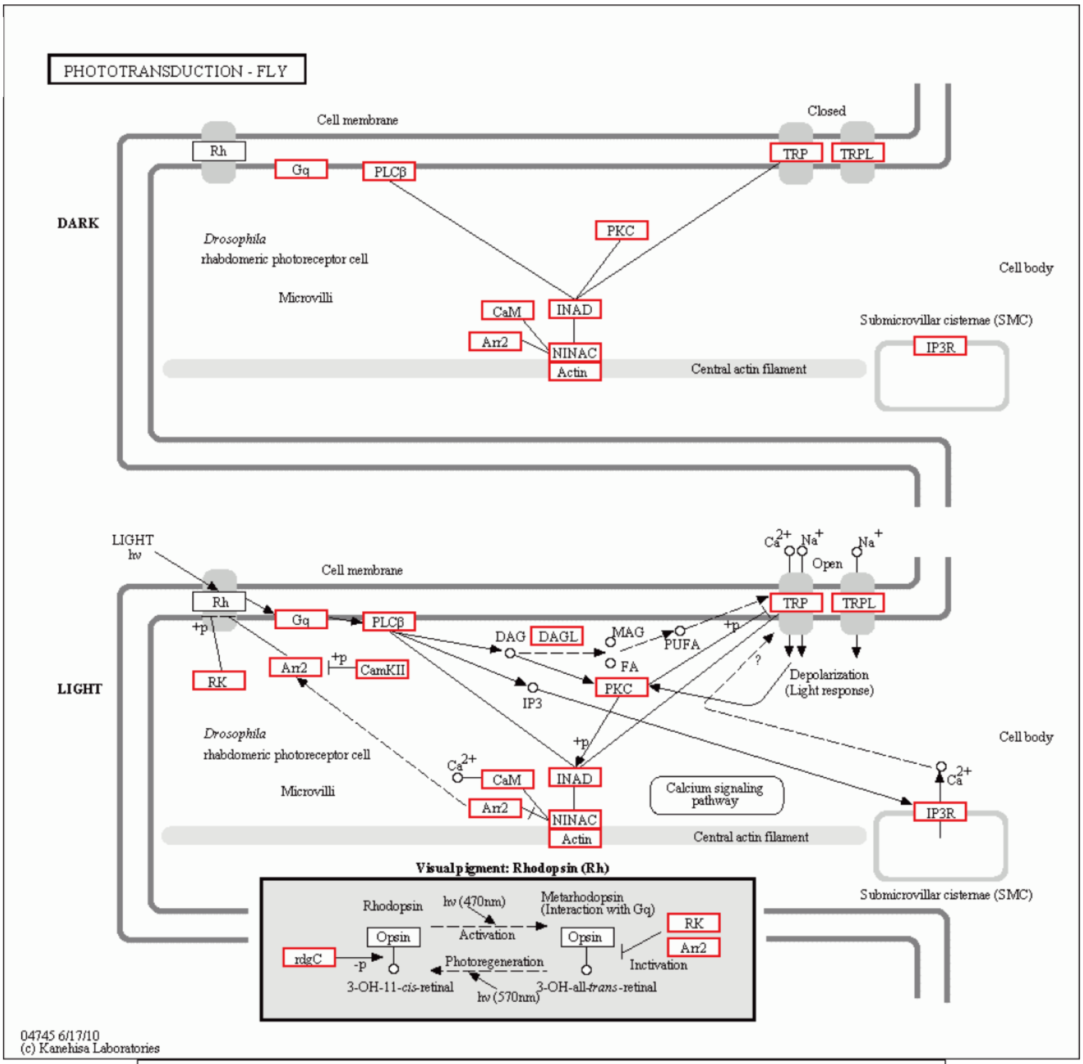

B

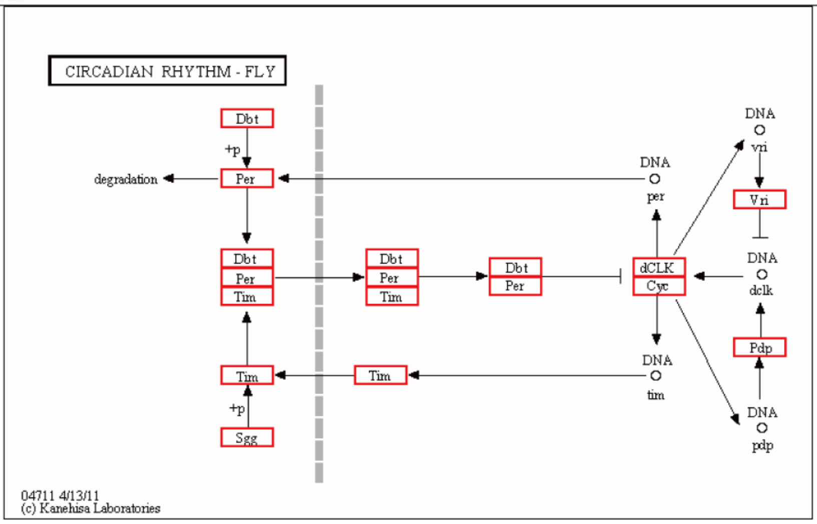

Figure S2. Phototransduction-fly pathway for unigenes by KEGG annotation (A) and circadian rhythm-fly pathway for unigenes by KEGG annotation (B).

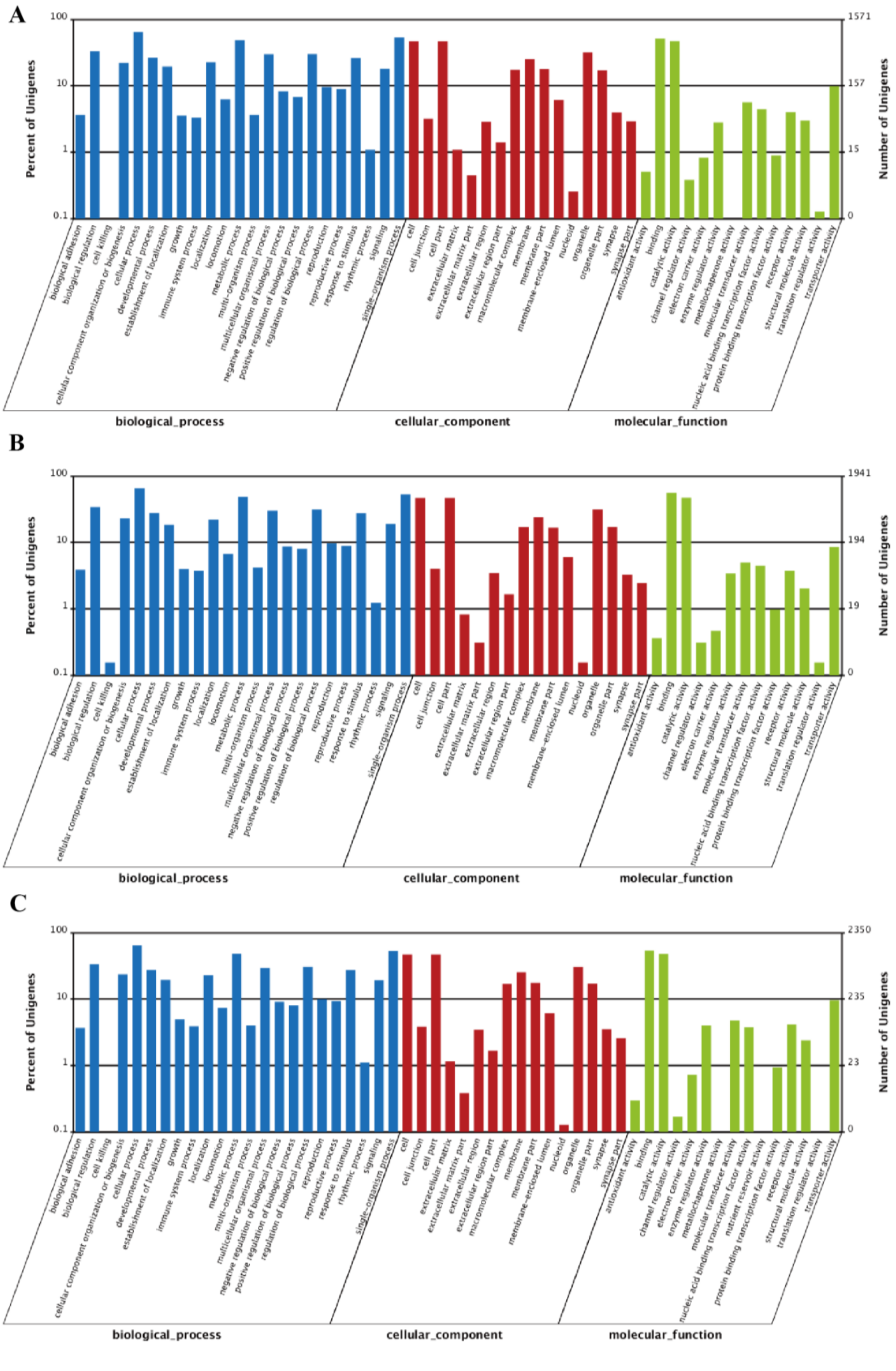

Figure S3. GO classification analysis of differentially expressed genes.

A: DEGs of dark vs white light; B: DEGs of dark vs UV light; C: DEGs of dark vs yellow light.

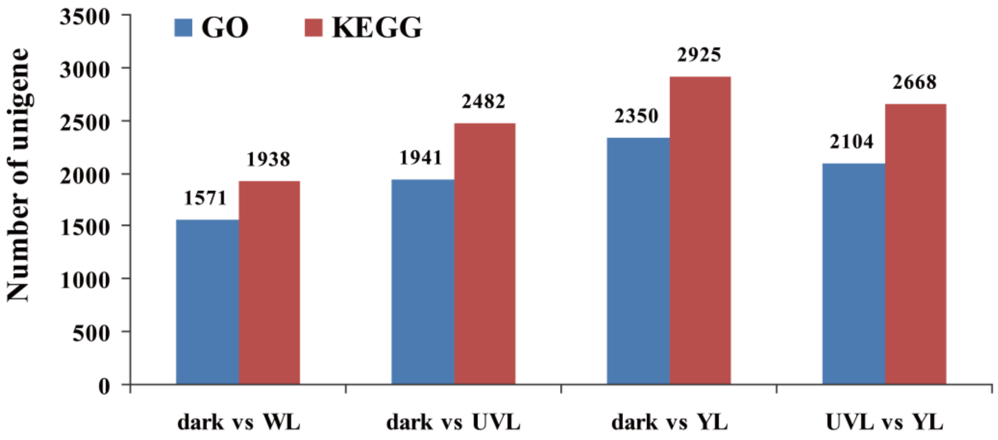

Figure S4. GO and KEGG classification analysis of differentially expressed genes.
